# Role of prion protein in mediating the synaptotoxic effects of tau oligomers: Implications for Alzheimer’s disease and related tauopathies

**DOI:** 10.4103/NRR.NRR-D-25-00516

**Published:** 2025-08-13

**Authors:** Roberto Chiesa, Luana Fioriti, Gianluigi Forloni, Claudia Balducci

**Affiliations:** Department of Neuroscience, Istituto di Ricerche Farmacologiche Mario Negri IRCCS, Milan, Italy

Alzheimer’s disease (AD) and other tauopathies are characterized by the accumulation of misfolded tau protein, which forms toxic oligomers that contribute to synaptic dysfunction and neuronal loss. Here, we briefly discuss recent findings indicating that the cellular prion protein (PrP^C^) plays a critical role in mediating the synaptotoxic effects of tau oligomers (TauOs), offering new insights into disease pathogenesis and potential therapeutic strategies.

**Tau oligomers as key mediators of synaptic dysfunction:** AD is the most common cause of dementia, characterized by progressive cognitive decline and neuronal loss. Pathologically, AD is defined by the accumulation of amyloid-β (Aβ) plaques and neurofibrillary tangles composed of hyperphosphorylated tau. While tau aggregates are a hallmark of AD and other tauopathies, it is increasingly recognized that soluble tau oligomers, rather than fibrillar tau, are the main drivers of synaptic dysfunction and neurotoxicity, as previously proposed for Aβ. These soluble tau species impair synaptic plasticity, reduce synaptic density, and disrupt neuronal communication, ultimately contributing to cognitive impairment (Spires-Jones and Hyman, 2014). However, the precise mechanisms by which tau oligomers exert their toxic effects remain a topic of active investigation.

**Prion protein as a mediator of tau toxicity:** Initially recognized for its role in prion diseases (Chiesa, 2015), PrP^C^ has since been implicated in a broader range of neurodegenerative disorders, including AD and Parkinson’s disease. In AD, the interaction between Aβ oligomers and PrP^C^ at the synapse appears to be a critical step in neurotoxicity (Um et al., 2012). This interaction leads to phosphorylation of the Fyn kinase via the metabotropic glutamate receptor 5, and subsequent phosphorylation of the N-methyl-D-aspartate receptor subunit 2B, leading to synaptic dysfunction in the hippocampus, impaired long-term potentiation (LTP) and destabilization of dendritic spines. Similarly, PrP^C^-mediated signaling has been proposed to contribute to synaptic dysfunction induced by α-synuclein oligomers in Parkinson’s disease (Ferreira et al., 2017). However, the validity and significance of these interactions have not always been consistently confirmed (Balducci et al., 2010; La Vitola et al., 2019).

Emerging studies have identified PrP^C^ as a receptor also for tau oligomers. It was found that the secretome of AD patient-derived induced pluripotent stem cells, containing soluble tau species, impaired LTP in rats, and this effect was prevented by antibodies targeting the N-terminal domain of PrP^C^ (Hu et al., 2018). Additionally, soluble tau aggregates, generated by sonicating recombinant tau fibrils, were shown to bind recombinant PrP^C^ via its N-terminal region. This form of tau disrupted neuritic integrity and impaired LTP in mouse hippocampal slices, and these effects were prevented by genetic ablation of PrP^C^ or pretreatment with anti-PrP^C^ antibodies (Corbett et al., 2020). While sonicated tau fibrils are shorter in size and biologically active, it remains unclear whether these assemblies represent bona fide oligomers or simply fragmented fibrils, which introduces some uncertainty regarding their structural identity.

**Figure 1:**
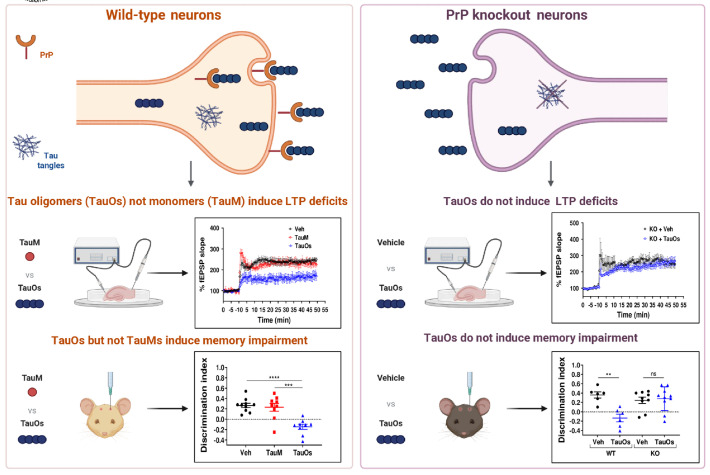
Cellular prion protein (PrP^C^) mediates the toxic activities of tau oligomers. Left: Tau oligomers (TauOs), but not monomers (TauM), inhibit long-term potentiation (LTP) in hippocampal slices and impair memory in PrP-expressing wild-type (WT) mice. Right: TauOs do not impair LTP and memory in PrP knockout (KO) mice. Adapted from Balducci et al. (2025). Created with BioRender.com.

We recently confirmed and extended these findings using recombinant TauOs generated from full-length human tau in the presence of arachidonic acid (Balducci et al., 2025; **Figure 1**). Surface plasmon resonance analysis found a dose-dependent, high-affinity interaction between TauOs and PrP^C^, with dissociation constant in the 20–50 nM range. In contrast, tau monomers showed no binding. Functionally, TauOs inhibited LTP in acute hippocampal slices from wild-type but not PrP^C^ knockout mice, whereas tau monomers had no effect. Furthermore, intracerebroventricular injection of TauOs significantly impaired recognition memory in wild-type, but not in PrP^C^ knockout mice, providing the first direct evidence of a PrP^C^-dependent effect of TauOs on cognitive function *in vivo*.

Immunofluorescence analysis of naïve and PrP^C^‐overexpressing HEK293 cells exposed to TauOs showed a PrP^C^ dose‐dependent association of TauOs with cells over time, as well as their co‐localization with PrP^C^ on the plasma membrane and in intracellular compartments, suggesting a role for PrP^C^ in TauO internalization. Together, these findings support the concept that PrP^C^ mediates the synaptotoxic effects of tau oligomers through a direct interaction. This mechanism may represent a shared pathway of neurotoxicity in AD and other tauopathies, offering novel therapeutic opportunities.

**Figure 2:**
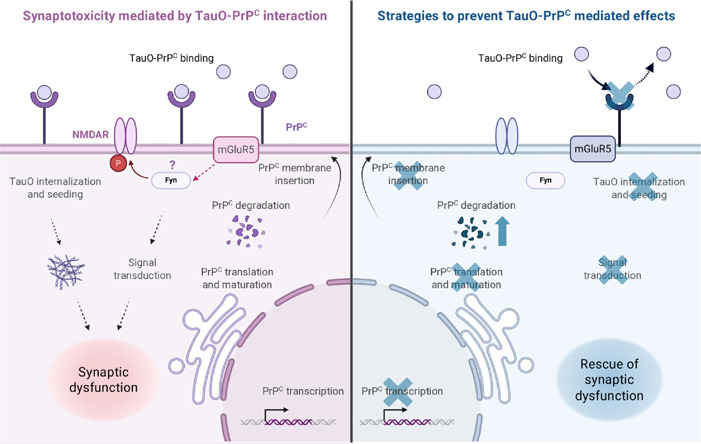
Strategies to prevent synaptotoxicity mediated by TauO-PrP^C^ interaction. Left: Schematic representation of the proposed synaptotoxic cascade triggered by the interaction between TauOs and PrP^C^, alongside the physiological maturation and processing of PrP^C^. Right: Overview of potential therapeutic strategies aimed at disrupting the TauO-PrP^C^ interaction, modulating downstream signaling pathways, or targeting PrP^C^ expression, trafficking, or processing. Created with BioRender.com. mGluR5: Metabotropic glutamate receptor 5; NMDAR: N-methyl-D-aspartate receptor; PrP^C^: cellular prion protein; TauO: tau oligomer.

**Targeting TauO-PrP**^**C**^ **interaction:** Given the emerging role of PrP^C^ in mediating the synaptotoxic effects of TauOs, disrupting this interaction, or inhibiting its effects, represents a promising therapeutic avenue. Several strategies could be explored as follows (**Figure 2**). (1) Blocking TauO-PrP^C^ binding: Small molecules, antibodies, or peptides designed to block the interaction between TauOs and PrP^C^ may prevent the toxic signaling triggered by this interaction. Since tau and Aβ oligomers appear to bind to overlapping sites on the N-terminal region of PrP^C^, targeting this domain could provide dual protection and maximize therapeutic efficacy. (2) Downregulating PrP^C^ expression: PrP^C^ suppression has no obvious deleterious consequences, and several therapeutic approaches aimed at reducing PrP^C^ expression in the brain are being explored in the context of prion diseases (Masone et al., 2025). Notably, anti-PrP^C^ antisense oligonucleotides have already entered clinical trials (https://clinicaltrials.gov/study/NCT06153966). These strategies could be repurposed or adapted to treat tauopathies, offering a novel means to mitigate TauO-driven neurodegeneration. (3) Modulating downstream signaling: PrP^C^ mediates Aβ oligomer toxicity through the metabotropic glutamate receptor 5, Fyn kinase, and Pyk2 kinase, and there is evidence that pharmacological inhibition of these pathways could offer neuroprotection (Haas et al., 2017). Whether targeting these pathways protects from TauO neurotoxicity too will require a detailed understanding of the TauO-PrP^C^ signaling (see below).

**Outstanding questions:** Despite recent advances, several key questions regarding the mechanisms of TauO toxicity and the role of PrP^C^ remain unresolved. First, the precise structural features of TauOs that interact with PrP^C^ require further elucidation. To date, most studies have used TauOs generated *in vitro* from recombinant tau protein. While these models offer experimental control, they may not fully recapitulate the structural complexity of pathological TauOs in human disease. It will be crucial to validate these findings using well-characterized TauOs purified from the brains of patients with AD and other tauopathies, such as frontotemporal dementia, progressive supranuclear palsy, and chronic traumatic encephalopathy. This approach would also allow investigation into the role of TauOs across different tauopathies, and help determine whether their neurotoxicity similarly depends on PrP^C^. If PrP^C^ is indeed a universal mediator of TauO toxicity, therapeutic strategies targeting this pathway could have broad implications for multiple neurodegenerative diseases. Conversely, if the TauO-PrP^C^ interaction proves to be disease-specific, understanding its mechanistic variability across tauopathies could guide the development of more tailored therapeutic interventions.

Second, it will be important to investigate the molecular detail of TauO-PrP^C^ interaction by biophysical methods and elucidate the downstream synaptotoxic signaling cascades. This will clarify both the similarities and differences in how PrP^C^ mediates the toxicity of different misfolded protein oligomers, including tau, Aβ, and α-synuclein, ultimately aiding in the design of tailored strategies to disrupt these harmful interactions.

Finally, there is growing experimental evidence that tau pathology in AD and related disorders spreads in a prion-like manner along anatomically connected brain regions. The observation that PrP^C^ may act as a cell surface receptor for TauOs suggests a potential role of PrP^C^ in tau spreading. Notably, PrP^C^ undergoes constitutive cycling between the plasma membrane and endocytic compartments, with a fraction of internalized PrP^C^ being recycled back to the cell surface. Our finding that PrP^C^ mediates TauO internalization in HEK293 cells (Balducci et al., 2025) supports the notion that endocytic recycling of the TauO-PrP^C^ complex may play a role in tau propagation. Interestingly, low-density lipoprotein receptor-related protein 1 has been implicated in clathrin-mediated internalization of cell surface PrP^C^ in neuronal cells (Taylor and Hooper, 2007), and has also been shown to mediate tau uptake and spreading (Rauch et al., 2020). Together these findings raise the intriguing possibility that the TauO-PrP^C^ complex may be internalized and recycled via low-density lipoprotein receptor-related protein 1, thereby contributing to tau propagation.

**Conclusions:** By facilitating tau oligomer binding and activating neurotoxic signaling pathways, PrP^C^ may contribute to synaptic dysfunction and disease progression in AD and other tauopathies. Disrupting the TauO-PrP^C^ interaction represents a promising therapeutic strategy, offering a novel means of mitigating tau-driven neurodegeneration. Future research should aim to validate the role of PrP^C^ in TauO toxicity in human-derived models and develop targeted interventions to interfere with this pathological axis.

A deeper understanding of the molecular mechanisms underlying TauO toxicity in relation to PrP^C^ could significantly advance the development of effective strategies for preventing and treating AD and other neurodegenerative conditions characterized by tau misfolding and oligomerization.

*This study was supported by the Italian Ministry of Health grant RF‐2021‐12372337 (to GF and CB).*

**Additional file:** *Open peer review report 1.*

OPEN PEER REVIEW REPORT 1
