# Supplementary material for: Role of prion protein in mediating the synaptotoxic effects of tau oligomers: Implications for Alzheimer’s disease and related tauopathies
Source: Neural Regen Res. 2025 Aug 13;21(8):3527–8. doi: 10.4103/NRR.NRR-D-25-00516 (PMC13452701; doi:10.4103/NRR.NRR-D-25-00516)
Supplement: Supplementary file 1 [file NRR-21-3527_Suppl1.pdf]

OPEN PEER REVIEW REPORT 1

Name of journal: Neural Regeneration Research
Manuscript NO: NRR-D-25-00516
Title: Role of prion protein in mediating the synaptotoxic effects of tau oligomers: Implications for Alzheimer's disease and related tauopathies
Reviewer's Name: Rodrigo Morales
Reviewer's country: USA

COMMENTS TO AUTHORS

This article comments on recent evidence suggesting a role of the cellular prion protein in the binding of tau oligomers. The article nicely introduces the subject and provides an appropriate comment to the previous work, as well as perspectives. I believe that some few changes and edits could improve the quality of this already nice article:

1. Several of the discussed evidence is based of using sonicated tau fibrils. Although shorter in size, it is unclear whether these aggregates represent bona fide oligomers or just fragmented fibrils. This limitation should be included.
2. It is suggested that the points listed in the "Targeting TauO-PrPC interaction" are included in the figure.
3. The final sentence in the last paragraph of the "Outstanding questions" section is not supported. It is suggested that this section is either merged with the following paragraph (that describes the topic in more detail). Another option is to just delete it as it is explained in the next paragraph.
4. If some of the data in the figure has been published, the reference should be cited in the legend.
